# Attitudinal Spillover from Misleading Natural Cigarette Marketing: An Experiment Examining Current and Former Smokers’ Support for Tobacco Industry Regulation

**DOI:** 10.3390/ijerph16193554

**Published:** 2019-09-23

**Authors:** Stefanie K. Gratale, Angeline Sangalang, Erin K. Maloney, Joseph N. Cappella

**Affiliations:** 1 Annenberg School for Communication, University of Pennsylvania, 3620 Walnut St., Philadelphia, PA 19104, USA; erin.maloney@asc.upenn.edu (E.K.M.); joseph.cappella@asc.upenn.edu (J.N.C.); 2 Department of Communication, University of Dayton, 300 College Park Ave., Dayton, OH 45469, USA; asangalang1@udayton.edu

**Keywords:** tobacco control policies, tobacco regulatory science, natural cigarette marketing

## Abstract

This research examined the influence of natural cigarette advertising on tobacco control policy support, and the potential for misbeliefs arising from exposure to cigarette marketing to affect such support. Ample research indicates that natural cigarettes such as Natural American Spirit (NAS) are widely and erroneously perceived as safer than their traditional counterparts because of their marketed “natural” composition. Yet regulatory action regarding natural cigarette marketing has been limited in scope, and little research has examined whether misleading product advertising affects support for related policy, an important component of the policy process. Here, we administered a large-scale randomized experiment (n = 1128), assigning current and former smokers in the United States to an NAS advertising condition or a control group and assessing their support for tobacco industry regulation. Results show that exposure to NAS advertising reduces support for policies to ban potentially misleading terminology from cigarette advertising, and these effects are stronger for daily smokers. Further, misinformed beliefs about the healthy composition of NAS partially mediate effects on policy support. Yet interestingly, exposure to NAS marketing does not reduce support for policies to establish standards for when certain terms are permissible in cigarette advertising. The results of this analysis indicate potential spillover effects from exposure to NAS advertising in the realm of support for regulatory action pertaining to tobacco industry marketing.

## 1. Introduction

Comedians have quipped that “organic” and “all-natural” are grocery store jargon for “double the price”. Yet for many people, these concepts represent tangible attributes of product healthfulness [1]—a dangerous proposition when potentially deadly products like cigarettes are marketed as natural or organic. Misleading product advertising has long been a concern for regulatory agencies [2,3,4,5], and when product advertising promotes inaccurate health information, regulatory policy becomes especially important for addressing related health behaviors [6,7]. In the present research, we aim to explore the attitudinal effects of health misinformation promoted by misleading advertising. Specifically, we explore the process in which potentially misleading natural cigarette advertising and the misbeliefs that can arise from it influence public support for policies to regulate such advertising. We specifically consider the marketing of Natural American Spirit (NAS), a leading natural cigarette brand.

Studies show that advertising for cigarette brands such as Natural American Spirit (NAS), which has been labeled as natural or organic for years, perpetuates erroneous beliefs that these cigarettes pose fewer health risks than their traditional counterparts [8,9]. Prior research evincing the harmful effects of misleading natural cigarette advertising has been cited as a rationale for potential regulation of cigarette marketing [10,11]. Despite the importance of policy action, little research has considered whether product advertising affects public support for related policy, an important component of the policy process. Thus, the purpose of the present research was to explore whether the effects of misleading advertising for products like natural cigarettes spill over into domains such as support for policies regulating product marketing. The present research focused on NAS marketing content available in the United States and may not represent the brand’s marketing worldwide, though the focus of the study included marketing terminology prevalent on the ads tested but also on NAS packs as well.

### 1.1. Concern Regarding Misinformation about Natural Cigarettes

Tobacco misinformation has permeated the information environment for decades [12,13], with tobacco companies having been found guilty of intent to mislead the public [14], and more recently, with misperceptions about natural cigarettes becoming especially prevalent. The images and text utilized in product marketing—such as that for natural cigarettes—may convey implicit assumptions about product healthfulness [15,16]. For instance, research has shown that packaging color, a “healthier” look and an organic label influence perceptions of food healthfulness, and such perceptions can extend to other types of organic products [17,18,19]. Research regarding natural cigarettes has tied the use of the word “natural” in ads or on packs to misbeliefs, as packs labeled natural, organic or additive-free are erroneously perceived as less harmful [9,10,20,21], especially among vulnerable groups including youth [22,23]. Analysis of NAS marketing has further supported these findings [24,25,26], showing that implicitly communicated misperceptions about NAS healthfulness influence intentions to use NAS [8,27]. Individuals who believe these health implications are more inclined to purchase NAS, and smokers frequently cite health concerns as a reason for choosing NAS [8,10].

Misbeliefs arising from natural cigarette advertising are particularly concerning because of the well-established effects of misinformation. Research about misinformation points to pernicious, lasting effects of debunked beliefs, as correcting misinformation is a complex, difficult process [28,29,30]. Even when it is seemingly corrected, misinformation can yield lingering effects on attitudes and related behaviors [31,32,33,34].

Misperceptions about natural cigarettes are especially problematic because misleading advertising may disproportionately influence current and former smokers, since they are particularly vulnerable to misleading health claims about cigarettes. Specifically, people are motivated to accept information and perspectives consistent with their own worldview, and this is particularly prevalent with regard to issues with which they are highly involved; individuals who are closely involved with an issue process issue-related information differently [35,36,37]. This can result in biased processing, motivated acceptance or rejection of information, and the maintenance of inaccurate perceptions [38,39,40,41,42]. As smoking is considered a defining trait of the self-concept, sometimes even among former smokers, smoker identity has been associated with outcomes including message processing as well as behaviors (e.g., rejection of anti-smoking messages, cessation attempts) [43,44,45,46,47]. Though policy and regulatory actions affect the general public, we are chiefly concerned with the opinions of smokers and former smokers regarding such policies. Because motivated reasoning makes current and former smokers especially vulnerable to misleading natural cigarette advertising—and misinformation arising from it is very difficult to correct—it is important that public policy measures address misinformation perpetuated by such advertising.

### 1.2. The Case for Regulatory Action

In cases of misleading advertising that influences health behaviors, regulatory policy can play an important role in addressing the public health threat, as such policy can lead to adjustment in health behaviors [6,7]. With regard to cigarettes, a relevant comparison arose with the advertising for cigarettes labeled as “light” or “mild”. Consumers incorrectly perceived these products as less harmful or less addictive, reducing their intentions to quit [48,49,50,51]. As the scientific evidence about misperceptions grew, the U.S. government acted to dispel misconceptions perpetuated by light cigarette marketing, through legislation prohibiting manufacturers from distributing tobacco products as light, low, or mild unless they had a Modified Risk Tobacco Product order [51]. Research suggests the removal of misleading package descriptors led to some decreases in public misperceptions about the safety of these products [52,53].

Like light/low-tar cigarettes, natural cigarettes are not considered reduced harm products [10,51,54]; yet commensurate regulatory action has not been taken regarding natural cigarettes. While public health advocates have called for a strong regulatory response, actions taken thus far have been limited in scope. For instance, natural cigarette packs must contain a warning label specifying that additive-free does not signify a safer cigarette [11,55]. Further, in 2017, Santa Fe Natural Tobacco Company (SFNTC, the manufacturer of NAS) reached an agreement with the U.S. Food and Drug Administration (FDA), the terms of which declared that SFNTC will refrain from using the words “natural” and “additive-free” in NAS marketing; however, the word “natural” will stay in the brand name, and SFNTC can incorporate related phrases such as “tobacco and water” and “organic” in NAS advertising [11,54,56]. Some research indicates that the agreement does not sufficiently address misinformation about NAS safety or composition [57]. Action thus far does not ban the specified terms from all cigarette advertising, and public health organizations maintain that it does not sufficiently address public health threats, highlighting the potential importance of additional policy action [54].

Public support for such regulation is relevant, both to the policy process itself and to subsequent health behaviors. Broadly speaking, public opinion is regarded as vital to the functioning of government and execution of policy that benefits the public interest [58,59,60,61,62]. Public opinion can serve, among other things, as an indicator of the need for and legitimacy of a policy [63,64,65]. Research in health areas such as alcohol control has shown that media coverage and public information about health issues affect public support for regulatory policy [63,66,67], and such support can thereby influence related regulatory decisions [68,69]. With regard to tobacco policy in particular, research indicates that public support may have an impact on policy implementation and success [69,70]. Regulatory policy, in turn, influences related public health behaviors [6,7]. As a result, considering the implications of natural tobacco advertising on public support for tobacco industry regulation is a valuable research avenue, and at present, an insufficiently examined one.

### 1.3. The Present Investigation

For the current research, we examined the influence of NAS advertising on tobacco control policy support, and the potential mechanisms of such influence. We aimed to test whether such advertising yields spillover effects on policy support, particularly when it prompts misbeliefs about NAS. The first goal was to explore effects of exposure to NAS advertising content on support for regulations banning certain terms from tobacco marketing. Specifically, we explored support for a ban of the terms “natural” and “additive-free” from tobacco advertising and packaging; these terms themselves have only been restricted to some extent by SFNTC’s voluntary agreement with the FDA, but they have not been banned and remain even in the brand name. Relatedly, we tested whether effects differed by smoker type, in line with expectations of motivated reasoning. Our final goal was to assess plausible mechanisms of effect, to ascertain whether inaccurate beliefs prompted by NAS advertising might influence policy support. Research shows that misbeliefs about the safety and healthfulness of NAS can mediate intentions toward NAS [27]. Here, we expected exposure to NAS advertising to influence policy support by prompting misinformed beliefs about NAS cigarette composition, as the policies in question pertain to restrictions on advertising language regarding the make-up of natural cigarettes (i.e., “natural”, “additive-free”). Our hypotheses were:

H1. Exposure to NAS advertising will reduce current and former smokers’ support for policies restricting permissible language in natural tobacco marketing.

H2. Effects of exposure to NAS advertising on policy support will be moderated by smoking status, and will be stronger among daily smokers.

H3. Misbeliefs about NAS cigarette composition will mediate effects on policy support among current and former smokers.

## 2. Materials and Methods

Our study was a randomized online experiment administered on the Qualtrics platform (analysis of different data from this sample was previously published [27]; that research explored the effects of NAS marketing on misbeliefs and intentions, but did not pertain to policy support).

### 2.1. Participants

This experiment included current and former adult smokers in the United States recruited by Qualtrics Panels, a national online survey panel provider. Current smokers had smoked at least 100 cigarettes and still smoked at the time of the study, and former smokers had previously smoked daily but had quit at least six months before participating [71]. We targeted a sample of current and former smokers based on the expectation of their susceptibility to misleading cigarette marketing, as well as the likely influence of motivated reasoning in their processing of NAS advertising. As specified, smokers may be especially inclined toward biased processing and motivated reasoning when encountering information pertaining to smoking; with regard to natural cigarettes, research indicates that smokers are especially vulnerable to misperceptions about brands like American Spirit, and smokers of the brand are more inclined to perceive it as less harmful [8,10,25].

### 2.2. Procedure

Participants completed this survey online via the Qualtrics platform using a computer (mobile devices were not allowed for this study). They first provided background information on demographics and smoking status; respondents not meeting age or smoking criteria were screened out of the survey. Eligible participants were randomized to a no-exposure control group or one of five treatment conditions. Following randomization, control group participants read instructions for the outcome measures and completed the measures. Participants in the treatment groups viewed two advertising stimuli randomly chosen from a set of three to four stimuli in their treatment condition (described in Section 2.3). After seeing stimuli for at least five seconds each, participants could then proceed to the next screen. Immediately following stimuli exposure, treatment group participants read instructions for the outcome measures and completed the outcome measures. At the end of the study, all participants received a debriefing message. All procedures received approval from the University of Pennsylvania Institutional Review Board.

### 2.3. Experimental Stimuli

Advertising stimuli were obtained from a search of NAS ads available through search engines or the NAS website. We categorized NAS stimuli into distinct treatment conditions based on the visual presentation format and textual word count of the advertisements. Four conditions utilized unedited NAS advertisements, and the fifth condition was a textual message using specific language taken from multiple NAS advertisements. The advertisements themselves were English language and represented both print and web marketing materials for the NAS brand. We did not hypothesize different effects by individual ad condition, but rather aimed to include a wide representation of nationally available NAS ad materials to which the public might be exposed via NAS marketing. We sought to broadly portray a range of NAS advertisements, and the claims made therein, in order to appropriately capture potential ad effects. Each condition had three or four stimuli in the available pool, of which participants in that condition saw a randomly assigned selection of two.

### 2.4. Measures

Our screener variables included age and smoking status. We also measured education, sex, race, ethnicity, preferred cigarette brand and whether participants had ever used natural cigarettes.

While our full data set included several categories of outcome measures, the focus of this study was support for policies regulating tobacco marketing in the United States. Specifically, our primary outcome measures assessed endorsement of “restriction” policies (n = 2, alpha = 0.92) banning the terms “natural” and “additive-free” from tobacco marketing and packaging. These policies would formalize the requirements in the FDA-SFNTC agreement into a rule applicable to all tobacco marketing. We also tested support for “oversight” policies (n = 3, alpha = 0.74) establishing standards for when such terms can be used, such as if a product has a scientifically demonstrated reduced risk; support for oversight policies should not necessarily be reduced as considerably by advertising exposure, and we did not hypothesize effects on these “oversight” policy variables. Policy measures used seven-point Likert agreement scales. Table 1 presents policy support measures, including individual measures as well as the scales respectively representing “restriction” and “oversight” policies.

While policy support was our primary outcome, we also included belief measures in the mediation analysis (described in “Data analysis”). Specifically, in our previous work cited above, we tested main effects of exposure to NAS marketing content on misbeliefs about NAS. In that analysis, we found that NAS advertising exposure triggers misbeliefs about the healthfulness and composition of NAS. For the present analysis, we were interested in mechanisms of influence on policy support. We used the belief scale regarding the composition of NAS as the mediator, since the policy measures pertained to statements about the makeup of NAS. Measures are available from the authors on request, and information about development of belief statements is available in our prior study.

### 2.5. Data Analysis

The first aim of this analysis was to assess effects of exposure to NAS advertising on support for tobacco control policy. One-way ANOVA with correction for multiple tests compared treatment means to the control group mean on individual and scaled policy measures. We also sought to identify whether effects were stronger for more “involved” participants. While we would optimally explore effects for NAS smokers, for whom motivated reasoning might apply most strongly, we only had 32 NAS smokers in our sample. We instead explored differences in effects for daily smokers (most “involved” with smoking), by testing the interaction of daily smoking and condition exposure. For moderation tests, we aggregated all treatment conditions, as we did not hypothesize differences in effect by condition, but rather an interaction of daily smoking status and exposure to any advertising condition vs. the no-exposure control. To probe for moderation, we utilized an independent samples *t*-test to establish differences in means on the “restriction” policy scale among daily vs. former or intermittent smokers exposed to any treatment condition; we then applied ANOVA to test for an interaction of daily smoking status and exposure to any treatment condition vs. control.

Our final goal was to assess possible mechanisms of effect. We expected that exposure to NAS advertising would lower support for restriction policies, in part by promoting misinformed beliefs about the healthy composition of NAS. To test this, we employed Hayes’ PROCESS model [72] for mediation. We used a dichotomous variable for exposure to treatment condition (vs. control) as the predictor, the composition belief scale as the mediator, and the “restriction” policy support scale as the outcome. As with the moderation analysis, we aggregated all treatment conditions in the mediation test because we did not expect different mechanisms of effect among individual ad conditions, but instead differences relative to control. All data analysis was conducted using the Statistical Package for the Social Sciences (SPSS) Versions 22–26. (IBM, Armonk, NY, USA)

## 3. Results

### 3.1. Participants

Our sample included 1128 current and former U.S. smokers. Five percent of current and two percent of former smokers designated NAS as their regular brand. The sample was 57% female; 87% white, 10% Black or African American, 6% other race; and 8% of Hispanic origin. Also, 47% of the sample indicated a high school degree or less, 22% had attended some college, and 31% had a college degree. In a soft launch, 72% of participants reported a college degree, atypical of our prior samples and the representative sample from the National Tips from Former Smokers campaign [73]; thus, we applied a quota of 35% for respondents holding a college degree for the full launch. The prior publication from this participant sample includes a breakdown of participant demographics and smoking status by condition.

### 3.2. Main Effects on Policy Support

Table 2 presents main effects of NAS advertising exposure on the individual and scaled policy support measures. Exposure to experimental conditions consistently resulted in reduced support for restriction policies banning the terms “natural” and “additive-free” from tobacco marketing, confirming Hypothesis 1. Yet, exposure to experimental conditions had minimal effects on support for policies to establish standards for when such terms may be used (oversight policies), and none that remained significant after a correction for multiple tests.

### 3.3. Moderation by Smoking Status

Our second hypothesis pertained to differences in effects based on smoking status. A *t*-test revealed that support for restriction policies was significantly lower among daily smokers than other respondents exposed to any treatment condition (mean difference = 0.49; *t*, 942 = -4.15, *p* < 0.001). ANOVA testing the interaction between treatment condition exposure (vs. control group) and daily smoking status provided qualified support for Hypothesis 2, showing that for daily smokers, exposure to experimental condition affected policy support more considerably, with a marginally significant interaction effect (*F*(1, 1124) = 2.83, *p* < 0.10). Table 3 displays mean results for the “restriction” and “oversight” policy scales, by smoking status (as with main effects, we observed no moderated effects with regard to the “oversight” policies).

### 3.4. Mediation Analysis

Mediation analysis tested the expectation that observed effects of condition exposure (vs. control) on policy support would be mediated by composition-related misbeliefs, as specified in Hypothesis 3. Analysis supported partial mediation, as the (significant) indirect effects accounted for a considerable portion of the total effect (see Table 4). We also tested an alternative hypothesis, in order to assess whether a plausible rival hypothesis could also explain the observed effects. Specifically, we explored whether observed effects might instead be mediated by attitudes towards NAS, which would not support our hypothesized path of effects. General attitudes towards the brand could theoretically affect other outcomes like policy support, but should not be as closely linked to misperceptions about NAS composition. Unlike the prior model, this model did not yield significant effects of exposure on the mediator (attitudes) or significant indirect effects; further, the effects of attitudes on policy support were much smaller than the effects of composition beliefs, thereby rendering general attitudes towards NAS a less plausible mediator of effects than our proposed mediator, misinformed beliefs.

## 4. Discussion

This study utilized an experimental design to assess the impact of exposure to NAS marketing on support for policies that restrict allowable terminology in natural cigarette marketing. We found that exposure to NAS advertising content lowers support for policies to ban the use of potentially leading terms such as “natural” and “additive-free” in cigarette advertising. Other work has established that advertisement exposure activates inaccurate beliefs about natural cigarettes, but the effect on policy support typically has not been examined. We were particularly interested in policy outcomes because ample prior research has shown that natural cigarette advertising perpetuates misinformation about the product, indicating a potentially important role for policies to regulate such marketing. Our data further show that the depressive effects of NAS advertising exposure on policy support may be partially mediated by the acceptance of inaccurate claims concerning the healthy composition of NAS cigarettes.

In particular, exposure resulted in consistent opposition for “restriction” policies that would ban the use of misleading terminology in tobacco advertising. Yet exposure bore no such effect on support for “oversight” policies, which focused only on establishing standards for when the terms can be used, such as when the product has a reduced risk. These findings are consistent with our expectation that exposure would depress support for policies that outright ban specific words from cigarette marketing; yet we would not expect the same opposition to policies that simply set forth guidelines for when such terminology is acceptable. The reason for this distinction is that we anticipated that inaccurate beliefs about the healthier composition of NAS resulting from advertising exposure would be a potential mediator of effects on policy support, at least to some degree. Specifically, respondents who are exposed to NAS advertising and believe the product to be of healthier composition than traditional cigarettes should have no inherent opposition to requiring that products must convey reduced risk in order to be called “natural” or “additive-free”; yet they should likely oppose an outright ban of such terms from the marketing of a product if they believe that product represents a safer, purer alternative to traditional cigarettes. Mediation analysis provided some support for this expectation, as inaccurate beliefs about the composition of NAS negatively predicted policy support and partially mediated effects of advertising exposure on such support. These results point to potential spillover effects of misperceptions perpetuated by NAS advertising, indicating that the misconceptions that engender the need for policy action serve to diminish public support for such action.

Moderation analysis offered qualified support for our hypothesis that effects would be stronger for daily smokers, in line with the expected role of motivated reasoning [74,75,76], as regular smokers have more motivation to focus on possible “benefits” of cigarettes when processing NAS advertisements [43,45,46]. These findings are concerning because the occurrence of motivated reasoning, likely enabled by misleading claims in NAS advertisements, may provide a justification for smokers to rationalize a knowingly dangerous behavior. Our knowledge of the persistent effects of misinformation [30], especially in cases of motivated reasoning in defense of one’s identity, further points to the potential value of more comprehensive policy action that can help to prevent the initial formation of inaccurate beliefs about the safety of natural tobacco. In fact, “the processes by which people form their opinions and beliefs are therefore of obvious public interest, particularly if major streams of beliefs persist that are in opposition to established facts” [30] (p. 107).

Our study had certain limitations. Our sample was more highly educated than a representative smoker sample [73]; still, this limitation is mitigated by the fact that the NAS smoking population tends to be more educated than the general smoking population [9]. In addition, our study included a sample of U.S. current and former smokers, and our stimuli included NAS marketing materials in English that could be available in countries that allow tobacco advertising. Our outcome measures also pertained to U.S. marketing policies. As a result, our results may not generalize to other countries with different policies governing tobacco advertising and with different exposure to NAS marketing. Nevertheless, it should be noted that some of the same terms in NAS advertisements also appear on the cigarette packs themselves, which could potentially engender similar effects.

Another limitation is the possibility that exposure to our stimuli might not accurately reproduce effects of exposure in a naturalistic setting. Specifically, participants completed the study on a computer and could have encountered distractions, and participation in an online study does not necessarily mimic incidental daily exposure to NAS marketing. Yet because of the limited exposure to two messages in the experimental context, our results likely only capture persuasive effects over and above the impact of naturalistic exposure to NAS advertising, thus rendering more consequential the significant effects observed with this limited exposure. Our mediation analysis was limited by the inability to make causal claims about the mediation pathway, as we did not manipulate the mediator; importantly, however, our analysis tested and yielded support for the a priori hypothesized pathway, yet it did not provide support for a different pathway using another potentially plausible (non-hypothesized) mediator.

Generally, prior research about natural cigarette advertising has shown that, much in the same way advertising for light cigarettes caused misconceptions about healthfulness, advertising for natural cigarettes gives rise to similar misconceptions. Still, research indicates the products do not convey reduced harm, though these inaccurate beliefs have been repeatedly established as a reason that smokers use the product. Our analysis has further shown that the mere existence of misleading natural cigarette advertising can yield other spillover effects, such as undermining public support for policies intended to combat these misperceptions. Public support is an important consideration in public policy, and health policies have been shown to be an effective means of improving public health by influencing health behaviors [6,7].

From a broader perspective, considering the unstated implications of tobacco advertising is relevant beyond the realms of light and natural tobaccos, with regard to new and emerging tobacco products. In June 2017 in the United States, for example, the FDA opened the public docket for commentary on its applications for Modified Risk Tobacco Products (MRTPs), which are products sold to reduce the risk of harm from traditional tobacco products [77]. As the FDA enters into new regulatory territory with regard to MRTPs, makers of these products will have to pass FDA scrutiny to obtain modified risk permits. Nevertheless, the potential for creating misleading beliefs through the framing of MRTP advertising (even in the absence of explicitly false claims) could emerge as a public health concern in this area. Thus, the current debate about policy regarding natural tobacco advertising is a timely and consequential one.

## 5. Conclusions

Our experimental findings indicate that exposure to NAS advertising reduces support for policies to restrict particular terminology from tobacco industry marketing. In fact, misinformed beliefs about NAS arising from brand advertising partially mediate effects on public support for policies intended to alleviate these misconceptions. Moreover, effects of the advertisements are somewhat stronger among daily smokers, who research shows are particularly susceptible to misleading natural cigarette marketing. In light of these results, and their relevance to nascent policies regarding MRTPs, our study underscores a potential role for more stringent policy action regarding the claims used in natural cigarette advertising. The insights gained from our examination of natural tobacco marketing should be further considered with regard to other tobacco products with labels that may convey misleading implications about the product.

## Figures and Tables

**Table 1:** Policy support measures.

Type of Measure	Measure Language
Restriction	Policy 1: The words “natural” and “additive-free” should be banned from tobacco advertising and marketing.
Restriction	Policy 2: The words “natural” and “additive-free” should be banned from tobacco product packaging.
Oversight	Policy 3: The FDA should establish some regulations for when tobacco companies can call their products “natural” or “additive-free” in advertisements.
Oversight	Policy 4: Tobacco companies should only be allowed to call their products “natural” or “additive-free” in advertisements if they can demonstrate the products are less harmful than traditional cigarettes.
Oversight	Policy 5: In order to include the words “natural” and “additive-free” in promotional materials, tobacco companies should have to provide scientific evidence that a product has reduced harm or risk.

**Table 2:** Mean (SD) comparison of treatment groups to control on policy support ^1^.

Measure	Control Group	Ad Condition 1 (graphic)	Ad Condition 2 (simple)	Ad Condition 3 (detailed)	Ad Condition 4 (web)	Ad Condition 5 (textual)
Policy 1	4.47	4.11	**3.96** **	***3.89*** **	***3.79*** ***	***3.61*** ***
	(1.52)	(1.83)	**(1.93)**	***(1.86)***	***(1.84)***	***(1.99)***
Policy 2	4.47	**4.05** *	***3.90*** **	***3.90*** **	***3.66*** ***	***3.62*** ***
	(1.60)	**(1.81)**	***(1.86)***	***(1.98)***	***(1.86)***	***(1.90)***
Restriction Scale (Policy 1, 2)	4.48	**4.08** *	***3.93*** **	***3.89*** **	***3.73*** ***	***3.61*** ***
	(1.45)	**(1.77)**	***(1.83)***	***(1.84)***	***(1.79)***	***(1.87)***
Policy 3	5.47	5.57	5.51	5.42	5.53	5.51
	(1.36)	(1.25)	(1.44)	(1.50)	(1.34)	(1.39)
Policy 4	5.23	5.22	**4.87** *	5.00	5.17	5.00
	(1.41)	(1.59)	**(1.72)**	(1.71)	(1.60)	(1.77)
Policy 5	5.65	5.58	5.36	5.39	5.39	5.44
	(1.35)	(1.36)	(1.66)	(1.61)	(1.52)	(1.52)
Oversight Scale (Policy 3, 4, 5)	5.45	5.45	5.25	5.27	5.36	5.32
	(1.09)	(1.15)	(1.27)	(1.35)	(1.27)	(1.18)

^1^ * *p* < 0.05, ** *p* < 0.01, *** *p* < 0.001; italics = *p* < 0.05 with Bonferroni correction; Note: Higher means indicate higher levels of policy support.

**Table 3:** Mean (SD) of treatment and control groups on policy support, by daily smoking.

Smoking Status	Control Group	Treatment Groups (Aggregated)
	Restriction Policy Scale	
Daily	4.10 (1.72) (n = 57)	3.62 (1.78) (n = 355)
Not Daily (former or intermittent)	4.07 (1.80) (n = 127)	4.11 (1.77) (n = 589)
	Oversight Policy Scale	
Daily	5.49 (1.19) (n = 57)	5.39 (1.23) (n = 355)
Not Daily (former or intermittent)	5.44 (1.14) (n = 127)	5.29 (1.24) (n = 589)

**Table 4:** Mediated effects of NAS advertising exposure on policy support.

Path	Coefficients (Standard Error) (Confidence Interval)
Effect of exposure on composition beliefs	0.39 *** (0.07) (0.25, 0.53)
Effect of composition misbeliefs on policy support	−0.63 *** (0.06) (−0.74, −0.52)
Total effects	−0.63 *** (0.14) (−0.91, −0.35)
Direct effects	−0.39 ** (0.14) (−0.66, −0.12)
Indirect effects	−0.24 *** (0.05) (−0.34, −0.16)

Note: ** *p* < 0.01, *** *p* < 0.001.
